# Preliminary validation of the paediatric vasculitis activity score (PVAS)

**DOI:** 10.1186/1546-0096-9-S1-O15

**Published:** 2011-09-14

**Authors:** Fiona E Price-Kuehne, Despina Eleftheriou, Seza Ozen, Michael Beresford, Pavla Dolezalova, Paul A Brogan

**Affiliations:** 1 Dept of Rheumatology, Institute of Child Health and Great Ormond Street Hospital, London, UK; 2 Hacettepe University Hospital, Ankara, Turkey; 3 Alder Hey Children’s Hospital, and University of Liverpool, UK; 4 Charles University 1st Medical Faculty, Prague, Czech Republic

## Background

There is a paucity of evidence-based data for the treatment of primary systemic vasculitis (PSV) in childhood, partly due to the lack of a standardised outcome-measure for use in clinical trials. The Paediatric Vasculitis Activity Score (PVAS) is a quantitative clinical-index of manifestations of active disease, divided into 9 organ sub-systems.

## Objective

To provide preliminary validation of the PVAS.

## Methods

Children at Great Ormond Street Hospital NHS Trust with a diagnosis of PSV underwent simultaneous assessment of disease activity by 2 assessors. Scores were assessed for inter-observer variability and correlation with the physician’s global assessment of disease activity (PGA), ESR and CRP. Patients with newly diagnosed PSV were assessed twice: at diagnosis and 1 month, to assess tool-responsiveness to a change in disease state.

## Results

23 children with PSV were studied - 48% male, 52% female. The diagnoses were: Behçet’s disease (n=7), Wegener’s granulomatosis (n=5), polyarteritis nodosa (n=5), cutaneous leukocytoclastic vasculitis (n=3), Cogan’s syndrome (n=1), microscopic polyangiitis (n=1) and unclassified vasculitis (n=1). Median PVAS was 1.5 (range 0-38). Bland-Altman analysis demonstrated high inter-observer agreement and Kappa analysis showed perfect agreement for 8/9 organ-system scores (K=1, p=0.00). Spearman’s rank showed correlation between PVAS and PGA (*r_s_*=0.87, 95%CI=0.71 to 0.94, p=0.00) and CRP (*r_s_*=0.54, 95%CI=0.10 to 0.81, p=0.02) but no correlation with ESR (*r_s_*= -0.1). Four newly diagnosed patients demonstrated a fall in the PVAS in response to therapy (change in median from 13.5/63 to 3.5/63), with good agreement for this change between assessors.

## Conclusion

This study provides preliminary evidence that PVAS is a useful clinical measure of vasculitis disease-activity with good inter-observer reliability and correlates highly with the PGA and CRP.

